# Combination of cephalosporins with vancomycin or teicoplanin enhances antibacterial effect of glycopeptides against heterogeneous vancomycin-intermediate *Staphylococcus aureus* (hVISA) and VISA

**DOI:** 10.1038/srep41758

**Published:** 2017-01-31

**Authors:** Chih-Cheng Lai, Chi-Chung Chen, Yin-Ching Chuang, Hung-Jen Tang

**Affiliations:** 1 Department of Intensive Care Medicine, Chi Mei Medical Center, Liouying, Tainan, Taiwan; 2 Department of Medical Research, Chi Mei Medical Center, Tainan, Taiwan; 3 Department of Internal Medicine, Chi Mei Medical Center, Liouying, Tainan, Taiwan; 4 Department of Health and Nutrition, Chia-Nan University of Pharmacy and Science, Tainan, Taiwan; 5 Department of Medicine, Chi Mei Medical Center, Tainan, Taiwan

## Abstract

Eight heterogeneous vancomycin-intermediate *S. aureus* (h-VISA) and seven VISA clinical isolates confirmed by the population analysis profile/area under the curve ratio (PAP/AUC) were collected. We further performed the PAP/AUC, time-killing methods and MIC tests using vancomycin/teicoplanin alone or combination with susceptible breakpoint concentrations of cefazolin, cefmetazole, cefotaxime, and cefepime for these isolates. The PAP/AUC MIC curve shifted left after addition of cephalosporins with vancomycin or teicoplanin for both h-VISA and VISA isolates. With the combination of different cephalosporins with vancomycin or teicoplanin, the AUC/Mu3 AUC ratio decreased to <0.9 for the standard Mu3 isolate which are compatible with the definition of vancomycin susceptible *S. aureus*. These decreases ranged between 1.81–2.02 and 2.37–2.85-fold for h-VISA treated with cephalosporins and vancomycin or teicoplanin, and 2.05–4.59, and 2.93–4,89-fold for VISA treated with cephalosporins with vancomycin or teicoplanin. As measured by time-killing assays, the combinations of different cephalosporins with vancomycin concentrations at 1/2 and 1/4 MIC, exhibited a bactericidal and bacteriostatic effect in VISA. The mean fold of MIC decline for vancomycin base combinations ranged from 1.81–3.83 and 2.71–9.33 for h-VISA and VISA, respectively. Overall, this study demonstrated the enhanced antibacterial activity of vancomycin/teicoplanin after adding cephalosporins against clinical h-VISA/VISA isolates.

Methicillin-resistant *Staphylococcus aureus* (MRSA) is a prevalent pathogen that causes human infections in community and hospital settings globally^1^,^2^,^3^. Moreover, MRSA infection can be associated with high morbidity and mortality^4^. Vancomycin is the most commonly used antibiotic to treat MRSA infections^4^,^5^,^6^. However, due to the increasing use of vancomycin, clinical MRSA isolates with reduced susceptibility to vancomycin have emerged recently^7^,^8^,^9^. Additionally, the clinical outcomes of heterogeneous vancomycin-intermediate *S. aureus* (h-VISA) and vancomycin-intermediate *S. aureus* (VISA) infections are poor^10^,^11^,^12^. Most important of all, the treatment options for h-VISA/VISA infections are limited^13^.

Aside from vancomycin, there are some viable alternative antimicrobial agents, such as daptomycin, linezolid, ceftaroline, trimethoprim/sulfamethoxazole, tigecycline, and quinupristin/dalfopristin^13^. Currently, these alternative choices with single antibiotics have not proved to be superior to vancomycin. Therefore, combination therapy may provide another option for combating this critical condition caused by h-VISA/VISA^14^,^15^. Werth *et al*.^15^ demonstrated vancomycin plus oxacillin or ceftaroline may improve the activity of vancomycin against h-VISA/VISA by enhancing vancomycin-cell wall interaction. Dilworth *et al*.^14^ showed the synergistic activity of vancomycin with piperacillin-tazobactam or oxacillin against VISA. However, no investigation has assessed the activity of combination of any of the four generations of cephalosporins with either vancomycin or teicoplanin against h-VISA/VISA. In this report, we conducted a comparative study of the combination of cephalosporins of all generations with either vancomycin or teicoplanin against h-VISA/VISA isolates by three laboratory methods to evaluate the *in vitro* antibacterial activity among different glycopeptide/cephalosporin (G/C) combinations.

## Material and Method

### Bacterial isolates

Eight h-VISA and seven VISA clinical isolates were collected from the Tigecycline *In Vitro* Surveillance in Taiwan (TIST) study, which collected clinical isolates from 22 hospitals between 2006 and 2010^16^. *Staphylococci* were identified by colony morphology, Gram stain, and coagulase test. MRSA isolates were further confirmed by the tube coagulase test and growth on 6 μg/ml oxacillin salt agar screen plates. Vancomycin MIC was measured by the agar dilution method. All h-VISA or VISA isolates were confirmed by calculating the population analysis profile/area under the curve ratio (PAP/AUC). Isolates were stored at −70 °C in Protect Bacterial Preservers (Technical Service Consultants Limited, Heywood, UK) until use. All eight h-VISA and seven VISA isolates were selected from different PFGE types, as previously described^17^,^18^.

### Antibiotics and MIC measurement

The MICs of cefazolin (CF), cefmetazole (CMZ), cefotaxime (CTX), cefepime (CPO), vancomycin (Sigma, St Louis, MO), and teicoplanin (Sanofi-Aventis, Bridgewater, NJ) were determined by agar dilution method, and interpretation criteria were based on the recommendations of the Clinical and Laboratory Standards Institute (CLSI)^19^. *S. aureus* ATCC 29213 was used as a control strain for MIC measurements.

### PAP/AUC for vancomycin and teicoplanin

The PAP/AUC was measured for all isolates by inoculating serial 10-fold dilutions of the test organism onto increasing concentrations of vancomycin- or teicoplanin-containing brain heart infusion (BHI) agar (Becton Dickinson, Sparks, MD, USA). The BHI agar plates contained vancomycin at the following concentrations 0, 0.5, 1, 1.5, 2, 3, 4, 6, and 8 mg/L or teicoplanin at the following concentrations 0, 1, 2, 4, 8, 16, and 32 mg/liter. Colony growth at 48 h was measured and graphed as log10 CFU/ml to obtain vancomycin PAPs (v-PAP) and teicoplanin PAPs (t-PAP). The v-PAP graph was used to calculate the AUC of each isolate, and the ratio of the AUC of the test isolate to the AUC of *S. aureus* Mu3 (ATCC 700698) was calculated. The AUC of tested isolate/Mu3 ratios were calculated. Ratios less than 0.9 were considered as VSSA and ratios of 0.9 to 1.3 and >1.3 were considered h-VISA and VISA, respectively^20^. Additionally, vancomycin criteria were extended to teicoplanin for this study.

### v-PAP/AUC and t-PAP/AUC reducing test

The PAP/AUC were detected with BHI containing vancomycin or teicoplanin alone and combination with susceptible breakpoint concentration of cephalosporins of all generations against eight h-VISA and seven VISA clinical isolates with the ratios of tested isolate/Mu3 were calculated. The fold decrease of vancomycin or teicoplanin alone with the G/C combinations were also calculated.

### Time-kill method

Eight h-VISA and seven VISA clinical isolates were selected for another *in vitro* measurement of inhibitory effect of combination regimens as recommended by the CLSI^21^. In brief, bacterial suspensions were diluted to 5.0 × 10^5^ colony-forming units (CFU)/mL in fresh Mueller–Hinton broth. Drug concentrations of vancomycin or teicoplanin were adjusted to 1/2xMIC, and 1/4xMIC. Each cephalosporin was used at susceptible breakpoint concentrations when in combination with a glycopeptide. Bacterial counts were measured at 8 h and 24 h by enumerating the colonies in 10-fold serially diluted specimens of 100 μL aliquots plated on the nutrient agar (Difco Laboratories, Sparks, MD) at 37 °C. All experiments were performed in duplicate.

Synergism was defined as a ≥2 log10 decrease in CFU/mL between the combination regimen and its most active constituent after 24 h and the number of surviving organisms in the combination regimen must be ≥2 log10 CFU/mL below the starting inoculum. In addition, at least one of the combination drugs must be present at a concentration that does not affect the growth of the test organism. Bacteriostatic and bactericidal activities were defined as <3 log10 and ≥3 log10 reductions in CFU/ml at 24 h, respectively, relative to the starting inoculum^21^.

### MIC change ratios of glycopeptide MICs

The MICs of vancomycin or teicoplanin alone and combined with 1x susceptible breakpoint concentration of a cephalosporin against eight h-VISA and seven VISA clinical isolates were determined by agar dilution method. A MIC ratio indicates the fold of the MIC decline of G/C combination *versus* a glycopeptide alone.

## Results

### The results of MIC tests

Table 1 shows the MICs of each cephalosporin, teicoplanin and vancomycin against eight h-VISA and seven VISA isolates. The MIC ranges of vancomycin and teicoplanin against h-VISA isolates were 1–2 mg/L, and 2–4 mg/L, respectively. For VISA isolates, the vancomycin MIC were all 4 mg/L, and teicoplanin MIC ranged from 4–16 mg/L. All of the h-VISA and VISA isolates were resistant to every cephalosporins based on the MIC level.

**Figure 1:**
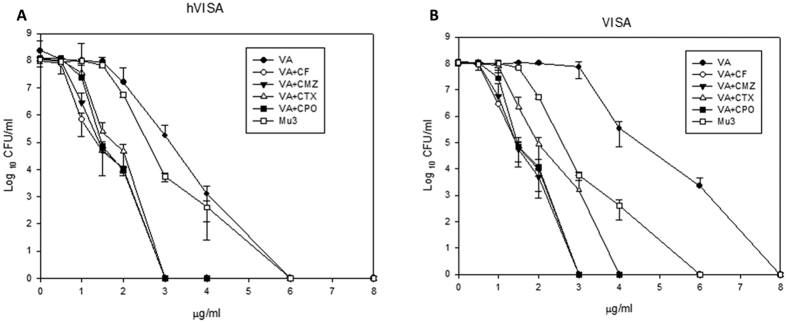
The PAP/AUC curve with vancomycin alone and in combination with susceptible breakpoint concentration of four cephalosporins against eight h-VISA (**A**) and seven VISA (**B**) clinical isolates with Mu3.

**Figure 2:**
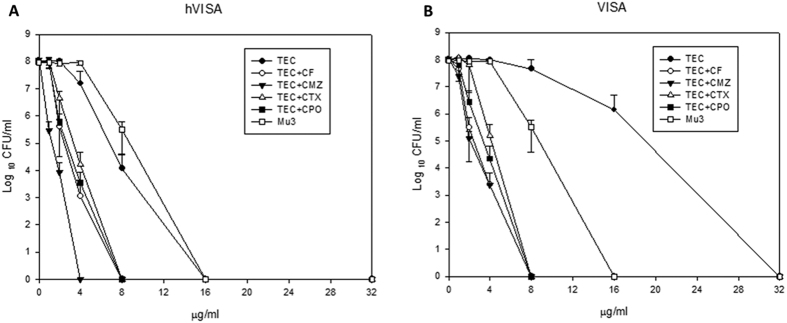
The PAP/AUC curve with teicoplanin alone and in combination with susceptible breakpoint concentration of four cephalosporins against eight h-VISA (**A**) and seven VISA (**B**) clinical isolates with Mu3.

Figure 1 shows that MIC curve shifts left after addition of different cephalosporins with vancomycin for both h-VISA and VISA isolates. Figure 2 shows the change of teicoplanin MIC against h-VISA/VISA after the addition of various cephalosporins. A similar phenomenon with vancomycin MIC was noted.

### PAU/AUC methods

The AUC/Mu3 AUC ratio of the eight h-VISA isolates were all approximately 0.95–1.14, which is compatible with the definition of h-VISA. In both hVISA and VISA and for all combinations of cephalosporins and vancomycin the PAP/AUC ratios decreased to below 0.9, compatible with VSSA. The v-PAP/AUC decreased between 1.81–2.02 and 2.37–2.85-fold after the combinations of cephalosporins for h-VISA and VISA isolates, respectively. For teicoplanin, the results were also the similar. With the combination of different cephalosporins with teicoplanin, all ratios decreased (<0.9), including the standard Mu3 isolate, and all of the tested h-VISA/VISA isolates are also compatible with the definition of VSSA. The t-PAP/AUC decreased between 2.05–4.59 and 2.93–4.89-fold after the combinations of cephalosporins for h-VISA and VISA isolates, respectively.

### Time-killing methods

Time-killing assays against each isolate are shown in Table 2. For h-VISA isolates, only one of the combination of vancomycin with CMZ exhibited bactericidal synergistic effect. One or two of the isolates disclosed bacteriostatic synergistic effect between other cephalosporins with vancomycin. Neither bactericidal nor bacteriostatic effect for the combinations of CTX was observed. For VISA isolates, such combination exhibited bactericidal synergistic effect against one to five of the seven tested isolates, respectively. Such combination also exhibited bacteriostatic synergistic effect against one to four of the tested isolates, respectively.

The *in vitro* activities of combination of CF, CMZ, CTX, CPO and teicoplanin were shown in Table 3. For h-VISA isolates, one to five of such combination exhibited bactericidal synergistic effect. One to four of the isolates disclosed bacteriostatic synergistic effect. For VISA isolates, such combinations exhibited bactericidal synergistic effect against five to six of the seven tested isolates. However, such combinations disclosed bacteriostatic synergistic effect against one to five of tested isolates, respectively.

### The changes of MIC levels

The MICs of vancomycin and teicoplanin with and without susceptible breakpoint concentration of cephalosporins for h-VISA/VISA isolates were shown in Table 4. The mean fold of MIC decline for vancomycin base combinations range from 1.81–3.83 and 2.71–9.33 for h-VISA and VISA, respectively. For teicoplanin base combinations, the mean fold of MIC decline range from 2.25–7.5 and 5.43–21.71 for h-VISA and VISA, respectively. The MICs of vancomycin and teicoplanin against h-VISA/VISA isolates lowered after combination with cephalosporins compared with single use. The decreases of MICs against h-VISA/VISA isolates seems to be highest for the combinations of CMZ and vancomycin or teicoplanin, followed by the CF regimen.

## Discussion

This study assesses the effect of addition of different cephalosporins with glycopeptides against h-VISA/VISA isolates and has several significant findings. First of all, we demonstrated the synergistic effect of glycopeptides and cephalosporins against h-VISA/VISA isolates by different methods based on this *in vitro* study. Initially, we used the PAP/AUC method to confirm both of the h-VISA and VISA isolates, and demonstrated the change of PAP/AUC curve to fit the criteria of VSSA from h-VISA/VISA after the combination of different cephalosporins. This study describes experiments using both vancomycin and teicoplanin. Secondly, the MICs of cephalosporins to h-VISA/VISA remained very high. However, when we used susceptible breakpoint concentrations which were far lower than MIC, the enhanced synergistic effect persisted even at 1/2, 1/4xMIC vancomycin or teicoplanin. Finally, via such combinations, we can also find the mean vancomycin and teicoplanin MIC decreases many folds. All of these findings indicated that even though h-VISA/VISA is insensitive to cephalosporins alone, combinations of antimicrobials and very low concentrations of cephalosporins still play important roles in the glycopeptide therapy of h-VISA/VISA.

However, previous studies^14^,^15^,^22^,^23^,^24^,^25^ had conflicting findings regarding the combined effect of glycopeptides and various antibiotics against h-VISA/VISA isolates. In Kim *et al*.’s report^22^, an antagonistic effect was observed in combinations with less than 1 μg/ml β-lactam antibiotics – oxacillin and cefotaxime combined with vancomycin, whereas synergistic effects were noticed in combinations with more than 4 μg/ml β-lactams antibiotics. In Aritaka *et al*.’s study^23^, they noted that when combined with vancomycin, four of the seven tested β-lactams, including ampicillin, oxacillin, imipenem, and cefmetazole, exhibited an additive effect at or near their MICs; however, in contrast, all of the tested β-lactams showed an antagonistic effect at lower sub-MIC levels. Therefore, their findings indicated that β-lactam antibiotics may not provide a significant advantage in combination with vancomycin against Mu3-like hetero-VISA strains. In contrast, we find the synergistic effect exist between cephalosporins and glycopeptides even at very low cephalosporin concentrations. The differences between the present work and previous investigations may be due to the different antibiotics we used for combination and different concentrations of antibiotics we used in this study.

In the report by Dilworth *et al*.^14^, four bloodstream VISA strains, the mean 24-h reductions in VISA inoculum for piperacillin-tazobactam, piperacillin-tazobactam with vancomycin, and oxacillin with vancomycin were 2.85, 2.93, and 3.45 log_10 _CFU/ml, respectively, and indicated the synergistic activity against VISA by vancomycin with piperacillin-tazobactam or oxacillin. In Leonard’s *in vitro* pharmacokinetic(PK)/pharmacodynamics(PD) study^25^, 23 (92%) of 25 h-VISA strains showed synergy with the combination of vancomycin and nafcillin in time-killing method, and among five selected strains demonstrated an improvement in overall activity and organism burden at 72 hours in PK/PD model. In Werth *et al*.’s study^15^ using time-kill assays, vancomycin plus oxacillin was synergistic against 3 of 5 VISA and 1 of 5 h-VISA isolates and vancomycin plus ceftaroline demonstrated a synergistic effect against 5 of 5 VISA and 4 of 5 h-VISA isolates. In Hanaki *et al*.’s study^24^, they found that teicoplanin showed the strongest synergistic effect in combination with imipenem, followed by, in decreasing order, panipenem, meropenem, flomoxef, sulbactam/ampicillin, cefoselis, and the average FIC indexes of the beta-lactam antibiotics against these strains were 0.113, 0.124, 0.163, 0.230, 0.264 and 0.388, respectively. All of the above findings suggest the synergistic effect of glycopeptide and beta-lactam combinations and are consistent with our result.

Although the different cephalosporin MIC values of tested h-VISA/VISA isolates remains high, the antibacterial effect was evident by a significant reduction of glycopeptide MICs, if serum achievable concentrations (1/2x or 1x SBC) of a cephalosporin were combined with sub-inhibitory concentrations of vancomycin or teicoplanin. Such an *in vitro* effect of glycopeptide MIC reduction was most obvious for cefmetazole among four tested cephalosporins. Therefore, although high cephalosporin resistance in clinical h-VISA/VISA isolates, we showed the strong evidence that cephalosporins can enhance the antibacterial activity of two commonly prescribed glycopeptides.

Several studies and meta-analyses have shown the significant association between vancomycin MIC and the outcomes of MRSA infections^26^,^27^,^28^,^29^. In Sakoulas *et al*.’s study^28^, vancomycin can successful treated for 55.6% of MRSA bacteremia while the MRSA isolates with vancomycin MICs < 0.5 μg/ml, but vancomycin was only effective for 9.5% of cases in which vancomycin MICs for MRSA were 1 to 2 μg/ml. For MRSA pneumonia, Haquel *et al*.^27^ found that the 28-day mortality could significantly increase while vancomycin MIC increased from 0.75 to 3 mg/mL (P ≤ 0.001). One recent meta-analysis of 2439 patients with MRSA infections (1492 high MIC (defined as ≥1 mg/l by BMD or ≥1.5 mg/l by E-test) and 947 low MIC) concluded that a high MIC to vancomycin is associated with increased mortality and treatment failure^26^. The similar influence of teicoplanin MICs on treatment outcomes among patients with teicoplanin-treated MRSA infection were shown in previous investigations and teicoplanin MIC ≥4 μg/mL (P = 0.0123) had a significant association with treatment failure in such patients^30^,^31^,^32^. In this *in vitro* study, we showed that vancomycin MICs of h-VISA and VISA isolates could be reduced to ≤0.75 μg/ml after combination with different cephalosporins, and even more, these combinations could make h-VISA or VISA become VSSA. For teicoplanin, its MIC of h-VISA and VISA isolates could also be reduced to ≤2 μg/ml after combining with cephalosporins. Although *in vitro* activity cannot represent *in vivo* effect, we can expect that the outcome improvement of h-VISA and VISA infections is possible to achieve after introduction of the glycopeptide/cephalosporin combination therapy based on our findings and previous investigations^26^,^27^,^28^. Of course, the application of such combinations to treat h-VISA/VISA infections needs more clinical investigations.

In conclusions, this is the first *in vitro* study by different methods to evaluate G/C combinations, and it showed the enhanced antibacterial activity against clinical h-VISA/VISA isolates, irrespective of cephalosporin MICs. Further comprehensive including *in vivo* study is warranted to investigate the combination effect between each class of antibiotics and glycopeptides.

## Additional Information

**How to cite this article:** Lai, C.-C. *et al*. Combination of cephalosporins with vancomycin or teicoplanin enhances antibacterial effect of glycopeptides against heterogeneous vancomycin-intermediate *Staphylococcus aureus* (hVISA) and VISA. *Sci. Rep.* **7**, 41758; doi: 10.1038/srep41758 (2017).

**Publisher's note:** Springer Nature remains neutral with regard to jurisdictional claims in published maps and institutional affiliations.

## Figures and Tables

**Table 1:** Minimal inhibitory concentrations (MICs) of four different cephalosporins and two glycopeptides against eight h-VISA and seven VISA clinical isolates.

	h-VISA								VISA							MIC breakpoints, mg/L		
	HV4	HV12	HV15	HV20	HV29	HV32	HV66	HV83	V2	V5	V6	V23	V31	V81	V82	S	I	R
CF	128	128	128	128	256	128	128	128	128	128	128	128	128	64	64	≦8	16	≧32
CMZ	64	64	64	128	128	128	128	64	32	64	64	64	64	64	64	≦16	32	≧64
CTX	512	512	1024	1024	1024	512	512	512	512	512	512	1024	512	512	512	≦8	16	≧32
CPO	64	128	128	128	128	128	128	128	64	128	128	128	128	64	64	≦8	16	≧32
TEC	4	2	4	4	2	2	2	2	4	8	16	8	8	16	16	≦8	16	≧32
VAN	2	2	2	2	1	2	2	2	4	4	4	4	4	4	4	≦2	4~8	≧16

Note. CF = cefazolin, CMZ = cefmetazole, CTX = cefotaxime, CPO = cefepime, TEC = teicoplanin, VAN = vancomycin.

**Table 2:** Eight h-VISA and seven VISA clinical isolates were selected for inhibitory effect of combination regimens at 24^th^ hour with 1/2 and 1/4 MIC of vancomycin and different cephalosporins of susceptible breakpoint concentration.

	Changes of colony count, log10 CFU/ml															
	CF 8 μg/ml				CMZ 16 μg/ml^a^				CTX 8 μg/ml^a^				CPO 8 μg/ml^a^			
	VA 1/2xMIC		VA 1/4xMIC		VA 1/2xMIC		VA 1/4xMIC		VA 1/2xMIC		VA 1/4xMIC		VA 1/2xMIC		VA 1/4xMIC	
	to start	to most active	to start	to most active	to start	to most active	to start	to most active	to start	to most active	to start	to most active	to start	to most active	to start	to most active
h-VISA																
HV4	−1.25	−4.17	2.92	0	−**2.08**	−**5.00**^c^	−0.63	−3.55	−1.48	−4.40	2.92	0	−1.88	−4.80	2.92	0
HV12	−0.44	−3.10	2.66	0	−0.50	−3.15	0.66	−2.00	−1.14	−3.80	2.66	0	−1.43	−4.09	2.66	0
HV15	−0.93	−4.11	3.18	0	0.04	−3.14	3.18	0	1.56	−1.62	3.18	0	2.56	−0.62	3.18	0
HV20	0.18	−2.52	2.70	0	−**2.62**	−**5.32**^c^	2.70	0	2.70	0	2.70	0	2.70	0	2.70	0
HV29	−1.00	−3.80	2.80	0	−1.79	−4.59	2.80	0	2.80	0	2.80	0	2.80	0	2.80	0
HV32	−0.18	−3.10	2.92	0	−0.38	−3.30	2.92	0	2.58	−0.34	2.92	0	−0.30	−3.22	2.92	0
HV66	−0.84	−3.49	2.66	0	−1.34	−4.00	2.66	−2.00	−0.40	−3.06	2.66	0	−0.76	−3.42	2.66	0
HV83	−**2.03**	−**5.08**^c^	3.05	0	−*3.95*	−*7.00*^b^	−**2.48**	−**5.52**^c^	−1.54	−4.59	3.05	0	−**2.07**	−**5.12**^c^	3.05	0
VISA																
V2	−*3.60*	−*5.30*^b^	−0.70	−3.49	−*3.43*	−*5.12*^b^	−*3.90*	−*6.70*^b^	−*3.06*	−*4.76*^b^	2.80	0	−**2.73**	−**4.43**^c^	1.72	−1.08
V5	−*3.05*	−*5.44*^b^	−0.95	−3.70	−*3.05*	−*5.44*^b^	−*3.00*	−*5.74*^b^	−*3.05*	−*5.44*^b^	1.22	−1.52	−**2.84**	−**5.23**^c^	−1.54	−4.28
V6	−*3.63*	−*5.70*^b^	−0.14	−3.21	−*3.93*	−*6.00*^b^	−*3.93*	−*7.00*^b^	−1.68	−3.74	3.07	0	−**2.35**	−**4.42**^c^	2.86	−0.21
V23	−1.40	−4.06	2.66	0	−*3.14*	−*5.80*^b^	−**2.20**	−**4.85**^c^	0.13	−2.52	2.66	0	−0.81	−3.47	2.66	0
V31	−1.49	−1.35	0.21	−2.44	−**2.43**	−**2.29**^c^	−**2.58**	−**5.24**^c^	−1.52	−1.38	1.86	−0.80	−1.59	−1.46	1.07	−1.59
V81	−**2.63**	−**4.48**^b^	−1.80	−4.49	−**2.44**	−**4.28**^c^	−**2.42**	−**5.12**^c^	−**2.12**	−**3.96**^c^	2.53	−0.17	−**2.27**	−**4.12**^c^	1.78	−0.92
V82	−*4.15*	−*4.20*^b^	−1.73	−4.59	−*4.15*	−*4.20*^b^	−*3.85*	−*6.70*^b^	−**2.94**	−**3.00**^c^	−0.80	−3.66	−*4.15*	−*4.20*^b^	−1.48	−4.34

^*^Cephalothin, CF; cefmetazole, CMZ; cefotaxime, CTX; cefpirome, CPO.

^a^Susceptible MIC breakpoints for MRSA isolates.

^b^Bactericidal with synergistic effect were showed in italic.

^c^Bacteriostatic with synergistic effect were showed in bold.

**Table 3:** Eight h-VISA and seven VISA clinical isolates were selected for inhibitory effect of combination regimens at 24^th^ hour with 1/2 and 1/4 MIC of teicoplanin and different cephalosporins of susceptible breakpoint concentration.

	Changes of colony count, log10 CFU/ml															
	CF 8 μg/ml^a^				CMZ 16 μg/ml^a^				CTX 8 μg/ml^a^				CPO 8 μg/ml^a^			
	TEC 1/2xMIC		TEC 1/4xMIC		TEC 1/2xMIC		TEC 1/4xMIC		TEC 1/2xMIC		TEC 1/4xMIC		TEC 1/2xMIC		TEC 1/4xMIC	
	to start	to most active	to start	to most active	to start	to most active	to start	to most active	to start	to most active	to start	to most active	to start	to most active	to start	to most active
h-VISA																
HV4	**−2.63**	**−4.48**^c^	**−**1.80	**−**4.49	**−2.44**	**−4.28**^c^	**−2.42**	**−5.12**^c^	**−2.12**	**−3.96**^c^	2.53	**−**0.17	**−2.27**	**−4.12**^c^	1.78	**−**0.92
HV12	**−***3.60*	**−***5.30*^b^	**−**0.70	**−**3.49	**−***3.43*	**−***5.12*^b^	**−***3.90*	**−***6.70*^b^	**−***3.06*	**−***4.76*^b^	2.80	0	**−2.73**	**−4.43**^c^	1.72	**−**1.08
HV15	**−***4.15*	**−***4.20*^b^	**−**1.73	**−**4.59	**−***4.15*	**−***4.20*^b^	**−***3.85*	**−***6.70*^b^	**−2.94**	**−3.00**^c^	**−**0.80	**−**3.66	**−***4.15*	**−***4.20*^b^	**−**1.48	**−**4.34
HV20	**−**1.40	**−**4.06	2.66	0	**−***3.14*	**−***5.80*^b^	**−2.20**	**−4.85**^c^	0.13	**−**2.52	2.66	0	**−**0.81	**−**3.47	2.66	0
HV29	**−**1.49	**−**1.35	0.21	**−**2.44	**−2.43**	**−2.29**^c^	**−2.58**	**−5.24**^c^	**−**1.52	**−**1.38	1.86	**−**0.80	**−**1.59	**−**1.46	1.07	**−**1.59
HV32	**−**1.28	**−**4.13	2.85	0	**−**1.48	**−**4.34	**−**1.45	**−**4.3	**−**0.77	**−**3.62	2.85	0	**−**1.31	**−**4.17	2.85	0
HV66	**−***3.05*	**−***5.44*^b^	**−**0.95	**−**3.70	**−***3.05*	**−***5.44*^b^	**−***3.00*	**−***5.74*^b^	**−***3.05*	**−***5.44*^b^	1.22	**−**1.52	**−2.84**	**−5.23**^c^	**−**1.54	**−**4.28
HV83	**−***3.63*	**−***5.70*^b^	**−**0.14	**−**3.21	**−***3.93*	**−***6.00*^b^	**−***3.93*	**−***7.00*^b^	**−**1.68	**−**3.74	3.07	0	**−2.35**	**−4.42**^c^	2.86	**−**0.21
VISA																
V2	**−***4.15*	**−***7.00*^b^	**−**1.41	**−**4.27	**−***4.15*	**−***7.00*^b^	**−2.50**	**−5.36**^c^	**−***3.24*	**−***6.10*^b^	2.85	0	**−***3.24*	**−***6.10*^b^	2.85	0
V5	**−***3.99*	**−***6.20*^b^	**−2.16**	**−5.32**^c^	**−***3.69*	**−***5.90*^b^	**−2.54**	**−5.70**^c^	**−***3.09*	**−***5.30*^b^	3.15	0	**−***3.21*	**−***5.43*^b^	**−**1.43	**−**4.59
V6	**−***3.79*	**−***7.00*^b^	**−**1.38	**−**4.59	**−***3.79*	**−***7.00*^b^	**−2.89**	**−6.10**^c^	**−***3.19*	**−***6.40*^b^	0.71	**−**2.49	**−***3.79*	**−***7.00*^b^	**−**1.86	**−**5.07
V23	**−2.82**	**−3.05**^c^	**−2.44**	**−5.36**^c^	**−***3.30*	**−***3.52*^b^	**−2.93**	**−5.85**^c^	**−**1.44	**−**1.66	2.92	0	**−2.48**	**−2.70**^c^	**−**1.55	**−**4.47
V31	**−2.22**	**−2.22**^c^	**−**1.40	**−**4.19	**−2.57**	**−2.57**^c^	**−2.03**	**−5.25**^c^	**−***3.60*	**−***2.90*^b^	2.60	**−**0.62	**−2.13**	**−2.13**^c^	**−**1.52	**−**4.32
V81	**−***4.20*	**−***3.00*^b^	**−**3.43	**−**6.22^b^	**−***4.20*	**−***3.00*^b^	**−***4.20*	**−***7.00*^b^	**−***4.20*	**−***3.00*^b^	**−2.60**	**−5.40**^c^	**−***3.90*	**−***2.70*^b^	**−***3.90*	**−***6.70*^b^
V82	**−***3.90*	**−***5.62*^b^	**−**3.60	**−**6.70^b^	**−***3.60*	**−***5.32*^b^	**−***3.90*	**−***7.00*^b^	**−***3.60*	**−***5.32*^b^	**−**1.28	**−**4.38	**−***3.90*	**−***5.62*^b^	**−2.90**	**−6.00**^c^

^*^Cephalothin, CF; cefmetazole, CMZ; cefotaxime, CTX; cefpirome, CPO.

^a^Susceptible MIC breakpoints for MRSA isolates.

^b^Bactericidal with synergistic effect were showed in italic.

^c^Bacteriostatic with synergistic effect were showed in bold.

**Table 4:** The MICs of vancomycin or teicoplanin in the absence and presence of susceptible breakpoint concentration of cephalosporins for h-VISA and VISA isolates.

	Control	CF, 8 μg/ml	CMZ, 16 μg/ml	CTX, 8 μg/ml	CPO, 8 μg/ml
**Vancomycin**					
h-VISA					
MIC range, μg/ml	1–1.5	0.75	0.38	0.75–1	0.5–1
Mean ± SD, μg/ml	1.44 ± 0.18	0.75 ± 0	0.38 ± 0	0.81 ± 0.11	0.81 ± 0.12
Mean fold of MIC decline	—	1.92	3.83	1.81	1.81
VISA					
MIC range, μg/ml	3–4	0.5–0.75	0.25–0.5	1–1.5	0.75–1.5
Mean ± SD, μg/ml	3.28 ± 0.49	0.71 ± 0.09	0.36 ± 0.05	1.29 ± 0.27	0.96 ± 0.27
Mean fold of MIC decline	—	4.76	9.33	2.71	3.67
**Teicoplanin**					
h-VISA					
MIC range, μg/ml	2–4	1	0.25–0.5	1–2	1–2
Mean ± SD, μg/ml	2.75 ± 1.04	1.00 ± 0	0.38 ± 0.13	1.25 ± 0.46	1.13 ± 0.35
Mean fold of MIC decline	—	2.75	7.50	2.25	2.50
VISA					
MIC range, μg/ml	4–16	1	0.5	2	1–2
Mean ± SD, μg/ml	10.86 ± 5.01	1.00 ± 0	0.5 ± 0	2 ± 0	1.86 ± 0.38
Mean fold of MIC decline	—	10.86	21.71	5.43	5.71

SD = standard deviation; cephalothin = CF; cefmetazole = CMZ; cefotaxime = CTX; cefpirome = CPO.
